# A 12-Year Longitudinal Case Report: Integrating Schema Therapy and Prolonged Exposure in Delayed-Onset PTSD Following the Great East Japan Earthquake

**DOI:** 10.1155/crps/9195824

**Published:** 2025-08-06

**Authors:** Arinobu Hori, Emiko Ando, Akihiko Ozaki, Michio Murakami, Masaharu Tsubokura, Fumiyo Oshima

**Affiliations:** ^1^Department of Psychiatry, Hori Mental Clinic, Minamisoma, Fukushima, Japan; ^2^Department of Neuropsychiatry, Fukushima Medical University School of Medicine, Fukushima, Fukushima, Japan; ^3^Breast and Thyroid Center, Joban Hospital of Tokiwa Foundation, Iwaki, Fukushima, Japan; ^4^Department of Thyroid and Endocrinology, Fukushima Medical University School of Medicine, Fukushima, Fukushima, Japan; ^5^Center for Infectious Disease Education and Research (CiDER), The University of Osaka, Suita, Osaka, Japan; ^6^EIPM Center, The University of Osaka, Suita, Osaka, Japan; ^7^Department of Radiation Health Management, Fukushima Medical University School of Medicine, Fukushima, Fukushima, Japan; ^8^Research Center for Child Mental Development, Chiba University, Chiba, Chiba, Japan

**Keywords:** posttraumatic stress disorder (PTSD), prolonged exposure therapy (PE), schema therapy, the Great East Japan Earthquake (GEJE)

## Abstract

**Objectives:** This case study examined the long-term course and treatment of posttraumatic stress disorder (PTSD) following the 2011 Great East Japan Earthquake (GEJE) and nuclear disaster. Specifically, this study investigated the role of early maladaptive schemas (EMSs) and coping modes in symptom persistence, the interplay between physical health issues and PTSD symptoms, and the efficacy of a staged treatment approach.

**Methods:** We present a 12-year longitudinal case study of a woman with delayed-onset PTSD. The patient underwent a staged treatment comprising supportive therapy, schema therapy, and prolonged exposure (PE) therapy. Treatment progress and symptom manifestations were qualitatively analyzed, focusing on schema modifications, coping mode changes, and trauma processing.

**Results:** The key findings were: (1) EMSs (e.g., enmeshment and subjugation) contributed to PTSD symptom maintenance and influenced postdisaster interpersonal patterns; (2) maladaptive coping modes played a role in symptom persistence and delayed disease onset; (3) the observed cyclical pattern of symptom exacerbation was particularly evident in the anniversary effect; (4) the staged treatment approach effectively addressed complex PTSD, with schema therapy facilitating subsequent trauma-focused interventions.

**Conclusion:** This study highlights the potential of combining schema therapy and PE to treat complex delayed-onset PTSD following compound disasters. This underscores the importance of addressing the underlying cognitive structures and coping mechanisms alongside trauma-focused interventions. These findings have implications for postdisaster long-term mental healthcare planning and suggest directions for future research to optimize treatment approaches for persistent PTSD.

## 1. Introduction

Posttraumatic stress disorder (PTSD) is a complex psychiatric condition characterized by significant long-term individual variability. A comprehensive meta-analysis by Ozer et al. [1] identified the key predictors of PTSD development, including prior trauma, prior psychological adjustment, family history of psychopathology, perceived life threats during trauma, post-trauma social support, peritraumatic emotional responses, and peritraumatic dissociation. These findings provide a foundation for understanding the risks and potential protective factors for PTSD development. Building on this, Santiago et al. [2] systematically reviewed PTSD prevalence and trajectories across various trauma-exposed populations, the results of which highlighted the heterogeneity of PTSD courses even within specific trauma types. The authors also emphasized the need for longitudinal studies better to understand the diverse pathways of PTSD development and recovery. Although numerous studies have suggested that PTSD symptoms tend to improve over time [3, 4], a substantial body of research indicates that some patients experience persistent or fluctuating symptoms over extended periods [5, 6 ]. Recent longitudinal studies have illuminated the diverse trajectories of PTSD, including late-onset cases [7], long-term persistence [8], and higher rates of avoidant symptoms and other variable courses in community samples [9]. These findings collectively underscore the complex and dynamic nature of PTSD, emphasizing the importance of considering both risk factors and long-term trajectories when understanding and treating the disorder. Recent developments in trauma diagnosis, particularly in the 11^th^ revision of the International Classification of Diseases (ICD-11), have introduced the formal diagnosis of complex PTSD, which is characterized by core PTSD symptoms along with additional disturbances in self-organization, including affect dysregulation, negative self-concept, and interpersonal difficulties [10]. The cognitive model proposed by Ehlers and Clark [11] provides a theoretical framework for understanding the persistence of PTSD symptoms, emphasizing the role of negative appraisals of trauma and its sequelae, as well as the nature of trauma memory in maintaining PTSD.

The 2011 Great East Japan Earthquake (GEJE), tsunami, and subsequent nuclear disaster provided a unique context for studying the long-term effects of compound disasters on mental health. Sakuma et al. [12] investigated the trajectories of PTSD symptoms among local disaster recovery workers following this event, identifying distinct patterns of symptom progression and highlighting the heterogeneity of PTSD courses even within a specific population exposed to the same traumatic event. To further our understanding of postdisaster mental health outcomes, Harigane et al. [13] examined psychological distress among residents of municipalities where the government issued evacuation orders after the nuclear accident. Their study, which was a part of the Fukushima Health Management Survey, identified problematic drinking tendencies, job loss, and social network size as three key factors correlated with high levels of psychological distress. These findings underscore the complex interplay among individual coping mechanisms, socioeconomic factors, and social support in shaping mental health outcomes after compound disasters. The mechanisms underlying variability in the course of PTSD remain insufficiently understood and require further investigation. We employed a qualitative case study methodology that allowed for an in-depth exploration of the complex interplay between psychological processes, life events, and symptom manifestations over time. This approach is particularly valuable for generating hypotheses regarding novel treatment approaches for complex long-term PTSD resulting from compound disasters. The 2011 compound disaster had profound and long-lasting effects on local communities, significantly complicating social and economic recovery efforts. This protracted recovery period is further exacerbated by subsequent challenges, including floods in 2019, the coronavirus disease 2019 (COVID-19) pandemic in 2020–2022, and major earthquakes (magnitude 7+) in 2021 and 2022. These continued adversities made it difficult for survivors to process their trauma and grief from the initial disaster fully; instead, they channeled their energy into community-rebuilding efforts. This societal pressure to focus on recovery activities, while psychologically adaptive in some ways, may have inadvertently delayed or complicated the processing of individual psychological challenges. Our case's initial presentation focused on physical health issues, specifically diabetes, with exacerbations that coincided with the anniversary of 2011 disaster. As treatment progressed, depressive symptoms and interpersonal difficulties became apparent, eventually revealing the underlying PTSD symptoms.

While trauma-focused therapies like prolonged exposure (PE) therapy [14] are supported to be effective for PTSD [15], their implementation in actual clinical settings is challenging, especially in postdisaster situations. Several factors contribute to this difficulty. First, these therapies are time-intensive for both patients and specialists. For instance, PE typically involves 90-min sessions conducted once or twice weekly, with a full course comprising 6–20 sessions. Additionally, therapists must be certified to administer these treatments. Second, trauma-focused psychotherapies inherently involve procedures that encourage patients to recall and confront traumatic memories. Without adequate therapeutic structures, these interventions could cause severe distress and potentially exacerbate symptoms.

PTSD is characterized by patients' attempts to avoid the distress associated with the activation of traumatic memories. This avoidance behavior serves a dual function: It temporarily alleviates the acute distress caused by PTSD symptoms while simultaneously impeding the processing of traumatic memories, thus, potentially prolonging the disorder. Trauma-focused psychotherapies, such as PE, are designed to address this paradoxical situation. However, some patients may still exhibit strong avoidance tendencies, anticipating the distress such treatments might evoke.

To overcome these therapeutic challenges, the authors posit that implementing schema therapy as an intermediary step between conventional supportive therapy and PE may prove beneficial. Schema therapy, an integrative psychotherapy approach developed in the 1980s and later, has demonstrated efficacy in treating individuals with chronic psychological problems [16–18]. The primary objective of schema therapy is to assist individuals in identifying and modifying long-standing patterns known as Early Maladaptive Schemas (EMSs). These EMSs are defined as “broad, pervasive themes or patterns comprised of memories, emotions, cognitions, and bodily sensations regarding oneself and one's relationships with others.” For example, the “Enmeshment/Undeveloped Self” schema is explained as the “excessive emotional involvement and closeness with one or more significant others (often parents) at the expense of full individuation or normal social development,” and the “Subjugation” schema is described as the “excessive surrendering of control to others because one feels coerced, submitting in order to avoid anger, retaliation, or abandonment” [16–18].

A key tenet of schema therapy is that EMSs are believed to have developed as defensive responses to traumatic experiences in childhood. Schema therapy integrates elements from cognitive behavioral therapy, attachment theory, psychodynamic concepts, and emotion-focused approaches by focusing on identifying and addressing core emotional needs that were unmet in childhood. This comprehensive therapeutic modality typically spans a duration of 1–3 years, allowing for in-depth exploration and modification of deeply ingrained patterns. The therapist should be certified [16–18].

In the context of PTSD treatment, particularly in cases where avoidance behaviors are pronounced, schema therapy would serve as a valuable preparatory phase. Addressing underlying EMSs and unmet emotional needs may facilitate patients' readiness to engage in more direct trauma-focused interventions such as PE. This staged approach potentially offers a more gradual and tolerable pathway for patients who might otherwise struggle with immediate engagement in trauma-focused therapies.

Our present study employed a staged approach incorporating supportive, schema therapies [16–18] and PE [14] to treat a PTSD patient who showed a compound course after the GEJE and nuclear accident. Typically, psychiatric outpatients receive standard treatment in conjunction with supportive psychotherapy. Within this framework, we administered schema therapy to patients presenting with trauma-related conditions, albeit without exhibiting acute PTSD symptoms such as intense re-experiencing. In cases where PTSD symptoms became manifest during schema therapy, we subsequently introduced PE. This approach allowed for the gradual development of trust between therapist and patient, carefully promoting habituation to approaching trauma memories, and achieving preparation through sufficient mastery of psychotherapeutic knowledge and techniques before implementing treatment specifically targeting intense PTSD symptoms.

This study offers a novel perspective on the long-term course of PTSD following a major compound disaster, integrating insights from cognitive models, schema therapy, and empirical trajectory studies while also considering the broader context of individual and social risk factors. By elucidating the potential role of defensive coping mechanisms in symptom maintenance and addressing the complex interplay between individual psychological processes and broader social factors, we hope to contribute to the development of more effective and personalized treatment strategies for individuals with persistent PTSD in the wake of complex disasters.

This case report was prepared in accordance with the CARE (CAse REport) guidelines [19]. The authors have completed the CARE checklist to ensure comprehensive and transparent reporting of the case.

## 2. Ethical Considerations

This study was conducted in accordance with the ethical standards of the 1964 Declaration of Helsinki and its subsequent amendments. The patient provided written and verbal informed consent for publication of this case report. All identifying information has been removed or altered to protect patient privacy.

## 3. Case Presentation

### 3.1. Patient

The patient is a female in her 40s.

### 3.2. Diagnosis

PTSD, diagnosed according to the criteria outlined in the Diagnostic and Statistical Manual of Mental Disorders, Fifth Edition (DSM-5) [20].

### 3.3. Life History

The patient grew up living with her grandmother, parents, and brother. When she was young, her parents were busy with their home-based businesses, and her grandmother was primarily responsible for her upbringing. Although financially comfortable, she often felt emotionally lonely. After graduating from vocational school, she worked in clerical positions at several companies. In her early 20s, she married and gave birth to two sons.

In March 2011, her husband died in the tsunami caused by the GEJE (Table 1). It took 2 months for her husband's body to be found, during which time she frequently visited the morgues to search for him. Her younger son became emotionally unstable after the earthquake and received counseling for 1 year. The younger son was diagnosed with thyroid cancer and underwent surgery for tumor removal. The patient and the other family members did not receive any psychological support.

Subsequently, the patient developed an overeating tendency. This tendency showed a notable annual exacerbation, with particular intensity observed each March, coinciding with the anniversary of the disaster. 1 year after the earthquake, the patient was diagnosed with diabetes and hypertension. In March 2019, 8 years after the earthquake, the patient's diabetes control worsened. The internal medicine physician determined that psychiatric treatment was necessary and referred the patient to our clinic. The first consultation was performed at our clinic in April 2019.

### 3.4. Treatment History

At the initial consultation, the patient spoke openly about her inner feelings, revealing that her diabetes control had consistently deteriorated each March for several years. She still had a strong aversion to the sea and avoided it. She had dreams of her younger son being swept away by the tsunami (which did not happen). She spoke about her husband's damaged body when it was found and expressed that she felt not only sadness but also anger, wondering “Why did he get caught in the tsunami and die, leaving us behind?”.

The patient was assessed as having PTSD with comorbid depression. This diagnosis was explained to the patient, along with the indication that intensive trauma-focused psychotherapy, such as PE, would be appropriate for her condition. However, the patient expressed reluctance to engage in such therapy and did not consent to begin it immediately.

The treatment team also judged this response to be understandable for several reasons: The patient exhibited significantly diminished vitality, making it difficult for her to engage in intensive psychotherapy at that time; her family members and colleagues were notably dependent on her, so cooperation from her environment for intensive treatment could not be expected; and, as part of the regional culture, there was a strong hesitation to speak publicly about one's trauma experiences.

Given these circumstances, the patient opted to begin treatment consisting of pharmacotherapy, brief supportive sessions, and instructions in breathing regulation techniques. Low-dose pharmacotherapy included oral tandospirone 20 mg/day. While the treatment staff recognized the severity of her condition, the patient reported feeling “calm” during consultations. When her eldest son developed hypertension, her anxiety and agitation increased, leading to an adjustment in her medication: Tandospirone was discontinued and replaced with sertraline 50 mg/day. However, her reported anxiety did not persist for long. In November of that year, when her residential area experienced flooding and the house suffered minor damage, she remained composed.

In March 2020, 9 years after the earthquake, the patient reported, “I had a few tsunami dreams. However, this year, there is more news about the COVID-19 pandemic and less coverage of the GEJE, which makes it easier.” 2 months later, approximately 1 year after starting treatment, she decided to quit her job, saying, “The work burden was too great, and my health deteriorated.” Upon resigning, she remarked, “It's strange how I've been choosing such demanding jobs since the earthquake.” This was the first time she had become aware of and consciously recognized her pattern of choosing excessively burdensome work after the disaster. The treatment staff suggested introducing more intensive psychotherapy; however, the patient was still reluctant to agree. Nevertheless, she said by August of the same year, “I'm okay with the earthquake now. However, I don't know what will happen as March approaches.” By the end of that year, the impact of COVID-19 pandemic had expanded to Japan.

In February and March 2021, the patient reported experiencing severe distress during another severe earthquake and expressed suicidal ideation. While the reduced media coverage of the GEJE caused by the COVID-19 pandemic provided some relief, she consciously avoided watching news programs about the disaster that year. In March, she participated in a local memorial service. The patient expressed anxiety when her eldest son, who had graduated from university, started his first job in April and found the work more demanding than they had anticipated. The patient resumed employment in June due to the termination of her unemployment benefits. Despite episodic increases in anxiety, she maintained her job while continuing to take sertraline, which was reduced from 50 to 25 mg/day in June 2021. That year, at the patient's request, the frequency of consultations decreased from once a month to once every 2–3 months. In December, her eldest son was diagnosed with depression, started psychiatric treatment, and took a leave of absence from work. Although psychologically distressed by her son's condition, the patient accepted a physically and mentally demanding temporary job from an acquaintance at the end of the year, resulting in generalized postcompletion pain. In January 2022, the frequency of consultations increased to once a month, and pharmacotherapy was intensified. Sertraline 25 mg/day was replaced with duloxetine 20 mg/day and trazodone 25 mg/day. In February, the dose of duloxetine was increased to 40 mg/day. This change in medication regimen led to temporary improvement.

During the March 2022 consultation, the patient made minimal reference to the disaster, despite having experienced another earthquake that month. However, in April, she reported experiencing daily nightmares. The authors once again explained the diagnosis of PTSD and emphasized the need for intensive trauma-focused psychotherapy.

By this stage, it had become clear that when the patient encountered difficulties or heightened anxiety, she tended to overextend herself in serving the needs of others, which in turn led to her own exhaustion. Accordingly, it was determined that in order for her to be able to confront her traumatic experiences, it was first necessary to modify these relational patterns and to prepare conditions in which she could preserve her own reserves of energy and prevent significant deterioration of her physical and mental health, even in the face of increased anxiety.

**Figure 1:**
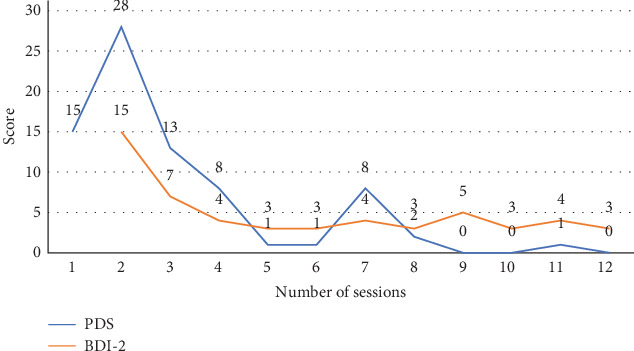
Symptom severity over treatment sessions. Posttraumatic Diagnostic Scale (PDS) measures PTSD symptom severity, shown in the blue line. Beck Depression Inventory II (BDI-II) measures depression severity, shown in the orange line.

In June, weekly cognitive behavioral and schema therapy sessions commenced. By April 2023 (12 years postdisaster, 4 years into treatment), 36 sessions had been completed. Subsequently, to address the prominent re-experiencing PTSD symptoms, treatment based on PE and psychotherapy for complicated grief was implemented from April to July, which included psychoeducation for PTSD, in vivo exposure, imaginal exposure, and processing. Over these 12 sessions, the patient's Posttraumatic Diagnostic Scale (PDS) [21] score decreased from 15 to 0, and the Beck Depression Inventory-II (BDI-II) [22] score from 15 to 3 (Figure 1). The PDS score was 15 immediately before initiating the PE-based treatment, increased to 28 before the second session, decreased to 13 before the third session, and gradually improved to 0. These assessments were conducted by the attending psychotherapist who was directly involved in the patient's treatment.

Subsequent monthly outpatient follow-ups were conducted. The patient increasingly reported an enhanced ability to resist excessive compliance with family expectations and to refuse when necessary. Positive changes were also observed in family dynamics.

In the New Year, 13 years postdisaster (5 years into treatment), when an earthquake occurred in another region of Japan, the patient initially experienced a brief period of incapacitation upon hearing the news. However, she recovered relatively quickly and expressed appreciation for the coping strategies acquired through the PTSD treatment.

The patient continued with outpatient consultations every 1–1.5 months and maintained stable employment. Her eldest son also improved following psychotherapeutic intervention and secured new employment, while the younger son continued his academic pursuits.

### 3.5. Content Implemented in Schema Therapy

Thirty-six schema therapy sessions were conducted from June 2022 to April 2023.

The introduction of psychotherapy focused on family issues especially about her eldest son, who suffered from depression, and her younger son, who required intensive psychological support after the earthquake. Furthermore, the patient's parents, who valued the tradition of favoring the eldest son, showed particular affection towards her eldest son, continuing this generational pattern. This kind of preferential treatment of the eldest son is a phenomenon observed in societies strongly influenced by confucianism in Asia, and it is still commonly accepted in rural areas of Japan even today. This familial dynamic, which prioritized her brother, contributed to the patient's emotionally unfulfilling and lonely childhood, characterized by a perceived lack of parental recognition and attention. The patient discussed her father, who ran a company, his physical decline, and his refusal to acknowledge this decline. She mentioned her family's tendency to prioritize public reputation and help others, even at the cost of burdening the family. Her father had a habit of telling her and her brother when they were small that “you were abandoned children we picked up,” seeming to enjoy their discomfort. Her parents were overly involved in the treatment of the patient's eldest son for depression. The authors pointed out that both the patient and her parents seemed to have an “enmeshment and undeveloped-self schema,” confusing concepts of identity and self-other boundaries, and discussed this. The patient's father had lost his father in the Pacific War and maintained a close relationship with his mother.

The authors learned about her experience of searching for her husband's body for 2 months after the GEJE, visiting morgues, and the state of her husband's body when found (his eyes and skin had melted after being trapped in a car and engulfed by the tsunami).

After starting psychotherapy, an incident occurred when the patient's younger son, who had poor academic performance, became agitated at home and required police intervention. The authors advised the patient to clarify the different roles and positions of parents and children, establish clear boundaries between individuals, and not comply with children's excessive demands. The patient understood and implemented the advice. Subsequently, she could refuse excessive demands from her children, parents, and friends outside the family. Her children, who had previously relied heavily on their mother, began to voluntarily share household chores. In schema therapy conceptualization, this process involved the patient understanding her own “subjugation schema” towards her parents and working to change it.

The patient realized that her overeating was a maladaptive “coping mode” to alleviate emotional impacts such as sadness and loneliness. After discussing coping modes in therapy, it became easier for her to recall and talk about her painful childhood experiences. She recalled memories of her mother sometimes acting coldly at home amidst the close relationship between her father and grandmother. By the 10^th^ session, she expressed surprise, saying, “I'm amazed at how much my childhood experiences are influencing my present.”

Amid recollections of lacking emotional support, memories of her connection with her husband were mostly happy and filled with love. After losing her husband to the tsunami, she became a single parent with two children. She internalized her father's words, “Children won't work if they don't see their parents working,” and reflected, “I think I was acting as if the wounds from the earthquake didn't exist.”

Schema therapy, in addition to “cognitive techniques” for understanding the above, also emphasizes “experiential techniques” to strengthen the image of healing one's inner wounded child and “behavioral techniques” to practice refusing in situations where one used to comply. In this patient's treatment, one of the authors developed sessions that deepened the cognitively recognized content experientially and behaviorally.

After the 17^th^ session, the patient was hospitalized for uterine fibroid surgery, resulting in a month-long interruption. Her two sons cooperated to overcome the challenges of maintaining and running the house. The patient reflected on how she might have been overly attached to her children, potentially hindering their development, and her behavior increasingly reinforced healthy boundaries with her sons. She found new employment in 2023, in a less physically and mentally demanding role. She also reported an increase in flashbacks related to earthquakes after the new year. The author concluded schema therapy after the 36th session in April 2023.

From there, we conducted 12 weekly sessions of PE to address the flashbacks related to the GEJE.

### 3.6. Content Implemented in PE

We initiated PE with psychoeducation on the mechanisms of exposure therapy and trauma responses. We introduced the concept of the Subjective Units of Distress Scale (SUDS) [23] to quantify subjective fear and discomfort. The patient then evaluated trauma-related locations, objects, and memories using the SUDS. We constructed an anxiety hierarchy to assess various trauma-inducing stimuli using the SUDS.

To promote habituation, in vivo exposure began with stimuli that elicit lower SUDS scores such as viewing earthquake-related videos on YouTube. At this stage, the patient remarked, “I couldn't have done this immediately. I think this was possible because I completed schema therapy first.”

Subsequently, we conducted imaginal exposure, which jointly recalled actual traumatic memories while monitoring the SUDS. The selected scenes were the moments of the GEJE and the confrontation with her husband's remains. During the initial implementation, while the patient did not report intense fear at a conscious level, she experienced severe abdominal pain necessitating a restroom break. She stated, “I'm in the habit of pretending to be fine.” The author identified the described status as the “emotional inhibition schema” from schema therapy.

Repeated imaginal exposure to the scene of touching her husband's remains revealed that grief over losing her husband was more challenging than fear. The patient expressed, “This sadness is the greatest sorrow of my life. That's why I am afraid of waves.” During processing, we addressed her thoughts and emotions to other family members related to her husband's death.

In the tenth session, we adapted our approach by incorporating the “imaginary conversation” technique drawn from complicated grief psychotherapy [24], in which the patient addressed her husband immediately after his death in a role-playing scenario. She said, “I wish you could have talked with the children, even for 5 or 10 min,” “I wanted to give you my lifespan,” and “Why do you make only me suffer so much?” In her imagination, her husband responded, “I'm sorry” and “It was bad timing.” She then reflected, “He must have wanted to see the children grow up too,” and declared, “This session's content was a bit challenging. I will overcome it and create stability at home.”

In the subsequent session, she reflected, “I didn't understand that my husband had died for 3–4 years. I started to feel it around the seventh year,” and “I realized it's been 12 years since my husband passed away.“ Subsequently, we conducted one session to review the entire psychotherapy process before concluding the intensive treatment.

After treatment, we continued to follow up with outpatient consultations approximately once monthly, with no significant issues arising.

## 4. Discussion

This case study provides significant insights into the long-term course of PTSD following the compound disaster of the 2011 GEJE and the subsequent nuclear incident.

### 4.1. Interaction of Physical Health Issues, Depressive Symptoms, and PTSD Symptoms

In the present case, physical health issues such as diabetes and hypertension, along with depressive symptoms, manifested before the direct expression of PTSD symptoms [25]. The patient's overeating tendency (coping mode) likely functioned as a form of emotional regulation that contributed to diabetes exacerbation.

The annual exacerbation of physical symptoms in march (the anniversary of the disaster) suggested a cyclical pattern of PTSD. This observation is crucial for understanding the temporal variability in the long-term course of PTSD [26].

The patient's employment pattern (tendency to choose excessively demanding jobs) can be interpreted as a manifestation of subjugation and enmeshment schemas. This can be understood as a maladaptive attempt to reconstruct self-worth and regain a sense of control after trauma [27].

### 4.2. Impact of the Staged Treatment Approach

We applied a staged treatment approach, progressing from supportive therapy to schema therapy, and finally to PE (Table 2). This staged approach appeared to be effective in addressing the complex symptoms of PTSD observed in this case.

Notably, schema therapy seemed to modify the patients' underlying EMSs and coping modes. This modification may have facilitated the acceptance and apparent efficacy of the subsequent PE. As the patient herself stated, “It was possible because I completed schema therapy first,” suggesting a potential link between treatment stages. However, this perceived relationship is based on the patient's subjective experience and our clinical observations and cannot be definitively established as a causal relationship in this single case study. Unlike in this case, when PTSD re-experiencing symptoms such as nightmares and flashbacks about traumatic memories are active, prioritizing treatments that directly target trauma symptoms like PE should be considered. Additionally, there may be cases where it is desirable to implement additional therapies, such as schema therapy, if other related psychological issues become apparent after completing trauma-focused therapy.

Regarding the course of treatment in this case, it is understandable that there could be criticism from the opposite perspective—namely, questioning why trauma-focused psychotherapy such as PE was not initiated earlier. In response to this, we wish to emphasize that PE presupposes weekly sessions lasting approximately 90 min each, conducted over 6–20 sessions. At the start of treatment, the patient's environment was characterized by pervasive dependance on her from those around her, and she had no reliable figures on whom she herself could depend. In this context, the patient, who was already severely depleted of vitality due to comorbid depression, would have faced significant difficulty in accepting a treatment likely to induce additional emotional turmoil.

As a prerequisite for initiating such intensive therapy, it was necessary to first modify the patient's interpersonal pattern of excessive service to others and enable her to accumulate sufficient emotional reserves to face her traumatic experiences.

The incorporation of techniques from complicated grief therapy, such as the “imaginary conversation” [24], appeared to be beneficial in addressing the patient's prolonged and intense grief reactions. This observation suggests the potential value of integrating grief-specific interventions in the treatment of trauma-related disorders following disasters involving the loss of life. However, further research is required to establish the efficacy of this combined approach in a larger and more diverse patient sample.

These observations provide a foundation for future research to more rigorously examine the potential benefits of a staged, integrative approach for treating complex PTSD following compound disasters. Controlled studies comparing this approach to other treatment modalities would be valuable in understanding the mechanisms and establishing the efficacy through which it may operate.

### 4.3. Consideration of This Case From the Perspective of Complex PTSD (ICD-11)

Although complex PTSD was not adopted in DSM-5, it is recognized as a formal diagnosis in ICD-11, and warrants important consideration. According to ICD-11, a diagnosis of complex PTSD requires exposure to an event or series of events of an extremely threatening or horrific nature, most commonly prolonged or repetitive events from which escape is difficult or impossible. Such events include, but are not limited to, torture, concentration camps, slavery, genocide campaigns, and other forms of organized violence, prolonged domestic violence, and repeated childhood sexual or physical abuse. In addition to core PTSD symptoms (re-experiencing, avoidance, and heightened sense of threat), complex PTSD requires the presence of disorders of self-organization (DSO), such as affect dysregulation, negative self-concept, and disturbances in relationships.

In our assessment, this case comes very close to meeting the ICD-11 criteria for complex PTSD, but ultimately does not fulfill them. One reason is that, aside from the earthquake and tsunami, the types of traumatic experiences did not include events that would typically be classified as involving life-threatening danger according to established PTSD diagnostic frameworks. While the patient experienced an upbringing marked by obligations to serve the family, was subject to capricious teasing by her father, and lived under significant postdisaster restrictions, these conditions, although traumatic and detrimental to core emotional needs as conceptualized in schema therapy, do not clearly meet the A-criterion of PTSD as defined in diagnostic systems like DSM [20].

Another consideration is that while the patient's excessive compliance with others' demands could potentially be viewed as a form of DSO, this pattern did not become evident until the severe social adversity following the disaster. Although we recognize that anxiety about “not fitting in” may have been latent, we find it difficult to conclude that enduring problems with affect regulation and relationships were present before the disaster. However, this is a provisional conclusion, and we believe that cases like the present one highlight areas where further research is needed. Moreover, this discussion indirectly underscores the importance of schema therapy, which targets EMSs and dysfunctional coping modes arising from unmet core emotional needs, regardless of formal diagnostic criteria.

### 4.4. This Patient's EMSs and the Long-Term Course of PTSD Symptoms

#### 4.4.1. Interpersonal Patterns and EMSs

Schema therapy identified this patient's EMSs, particularly the “enmeshment” and “subjugation” schema [16–18, 28]. These schemas fostered an environment in which colleagues and children were excessively dependent on the patient, leading to her exhaustion. Notably, these patterns originate from childhood relationships with her parents and persist into adulthood until treatment commencement.

By recognizing and modifying these patterns through schema therapy, patients develop the ability to establish healthy boundaries and refuse excessive demands [16–18]. This change reduces patient fatigue and creates space for further therapeutic progress. This process corroborates the importance of modifying post-trauma maladaptive beliefs and behavioral patterns, as emphasized in Ehler and Clark's [11] cognitive model of PTSD.

#### 4.4.2. This Patient's Maladaptive Coping Modes and PTSD Symptom Maintenance

The patient's overeating and excessive care for others were interpreted as maladaptive “coping modes” in Schema Therapy terminology. Schema therapy allowed the patient to recognize and bypass the defensive functions of these coping modes, enabling her to confront and process her feelings of hurt, sadness, loneliness, and anger. This exemplifies the central therapeutic mechanism of schema therapy, the efficacy of which was demonstrated in this case [16–18]. Although coping modes may provide short-term symptom relief, they can impede the proper processing and integration of traumatic memories and the grief in the long term, leading to symptom persistence and fluctuation. This observation provides important insights into the mechanisms underlying symptom variability and delayed onset during the long-term course of PTSD [7–9], and aligns with Harigane et al.'s [13] findings on the importance of modifying maladaptive coping strategies, such as problem drinking. Furthermore, their research indicated associations among job loss, social network size, and levels of psychological distress, suggesting that improvements in patients' interpersonal patterns and employment situations may have contributed to symptom improvement.

### 4.5. Cultural and Contextual Influences on Schema Formation and PTSD Course

The formation of EMSs and maladaptive coping modes can be partially attributed to the cultural influences of Japanese society. These cultural factors include the valorization of modesty in self-expression and the expectation of fulfilling the roles prescribed by the elderly, family, and community. The hierarchical nature of Japanese society, which often manifests in age-based seniority systems and gender disparities, reinforces these schemas. This is further evidenced by Japan's consistently low ranking in the Global Gender Gap Index, highlighting significant gender inequalities in various aspects of society [29]. Additionally, the cultural emphasis on maintaining social standing often takes precedence over nurturing intimate family relationships, potentially contributing to enmeshment and subjugation schemas

The cultural expectation of prioritizing social harmony and community needs over individual emotional rest after disasters may have contributed to the delayed onset and prolonged course of PTSD symptoms. Moreover, the cultural stigma associated with mental health issues might have deterred the patient from seeking professional help, thereby, contributing to the delayed diagnosis and treatment. Even when the internal physician treating diabetes recognized the existence of mental health issues, they may have delayed referring the patient to a psychiatrist out of consideration for the patient's feelings. Understanding these cultural and contextual influences is crucial for understanding the complex interplay between individual psychopathology and societal factors in the development and maintenance of PTSD following compound disasters.

### 4.6. Limitations

Although this single case study provides valuable insights into the long-term course and treatment of PTSD following a compound disaster, several limitations must be acknowledged:

1. Generalizability: Patients' unique circumstances, including cultural background, family dynamics, and the specific nature of the compound disaster, may not be representative of all individuals with PTSD. However, the detailed exploration of schema formation, coping modes, and staged treatment approach offers important hypotheses for future research.
2. Retrospective analysis: The retrospective nature of this study, particularly in analyzing the long-term course of PTSD, may have been subject to recall bias. While we attempted to corroborate the information from multiple sources (patient reports, clinical observations, and standardized measures), some details may have been influenced by the patient's current status or reconstructed memories.
3. Lack of controls: As a single case study, we cannot definitively attribute improvements solely to treatment approaches. Other factors, such as natural recovery processes, changes in life circumstances, or external support systems, may have contributed to the observed improvements.

## 5. Conclusion

This case study demonstrates the role of EMSs and coping modes in the long-term course of PTSD following a compound disaster, the complex interplay between physical and psychological symptoms, and the efficacy of a staged, individualized treatment approach. These findings have important implications for the planning and implementation of long-term mental healthcare after compound disasters.

In other words, the EMSs formed over the course of the patient's life significantly influence the way the patient adopts avoidance strategies to cope with the distress caused by PTSD symptoms. The EMSs and maladaptive coping modes employed as avoidance strategies shape the patient's interpersonal relationships, work habits, and interactions with society after the disaster. This creates an environment that, in turn, influences the course of the patient's PTSD. Conversely, this case suggests that by identifying and intervening in the specific EMSs and coping modes that are activated in a particular patient, it may be possible to positively alter the course of PTSD.

Future research is needed to validate these observations in larger samples and explore the factors that explain individual differences in PTSD trajectories following compound disasters.

## Figures and Tables

**Table 1:** Case timeline: symptoms and treatments from 2011 to 2024.

• March 2011: Great East Japan Earthquake, tsunami, and husband's death
• May 2011: Husband's body found
• March 2012: Diagnosed with diabetes and hypertension
• March 2019: Worsening of diabetes and referral to psychiatry
• April 2019: Start of treatment at our clinic (supportive therapy and pharmacotherapy)
• November 2019: Flooding
• March 2020: Impact of the COVID-19 pandemic
• May 2020: “Realization of having chosen and engaged in demanding jobs after the earthquake” and “Decision to resign.”
• February–March 2021: Another earthquake and suicidal ideation
• April 2021: Her son's employment
• December 2021: “Son's diagnosis of depression” and “Taking on a demanding temporary job.”
• March–April 2022: Another earthquake and manifestation of PTSD symptoms
• June 2022: Start of schema therapy
• April 2023: End of schema therapy (36 sessions)
• April–July 2023: PE (prolonged exposure) therapy (12 sessions)
• January 2024: Follow-up and maintenance of symptom improvement

**Table 2:** Key distinctions between two evidence-based trauma therapies.

Treatment characteristic	Prolonged exposure therapy (PE)	Schema therapy
Primary treatment target	Acute trauma symptoms and specific traumatic memories	Core beliefs and persistent behavioral patterns developed from life experiences
Therapeutic strategy	Systematic desensitization through controlled memory recall and situational exposure	Multimodal intervention combining cognitive restructuring, emotional techniques, and behavioral modification
Treatment considerations	Most suitable for distinct traumatic events; may not address deeper personality structures	Comprehensive but requires significant time investment; less direct focus on immediate trauma symptoms
Case focus	Trauma from encountering a deceased husband's body and grief	Enmeshment and subjugation schemas: Pattern of excessive accommodation to others' expectations, causing others to become dependent on the patient resulting in increased physical and psychological burden

## Data Availability

Data sharing is not applicable to this article as no new data were created or analyzed in this study.
